# Ultrafast measurements of optical spectral coherence by single-shot time-stretch interferometry

**DOI:** 10.1038/srep27937

**Published:** 2016-06-13

**Authors:** Yiqing Xu, Xiaoming Wei, Zhibo Ren, Kenneth K. Y. Wong, Kevin K. Tsia

**Affiliations:** 1 Department of Electrical and Electronic Engineering, The University of Hong Kong, Pokfulam Road, Hong Kong

## Abstract

The palette of laser technology has significantly been enriched by the innovations in ultrafast optical pulse generation. Our knowledge of the complex pulse dynamics, which is often highly nonlinear and stochastic in nature, is however limited by the scarcity of technologies that can measure fast variation/fluctuation of the spectral phase (or coherence) and amplitude in real-time, continuously. To achieve this goal, we demonstrate ultrafast interferometry enabled by optical time-stretch for real- time spectral coherence characterization with microsecond-resolution. Accessing the single-shot interferograms continuously, it further reveals the degree of second-order coherence, defined by the cross-spectral density function, at high speed-a capability absent in any existing spectroscopic measurement tools. As the technique can simultaneously measure both the high-speed variations of spectrally resolved coherence and intensity, time-stretch interferometry could create a new arena for ultrafast pulse characterization, especially favorable for probing and understanding the non-repetitive or stochastic dynamics in real-time.

Ultrafast pulsed lasers continue to advance a wide range of disciplines, from frequency metrology, telecommunication, biochemical spectroscopy, to optical imaging^1^,^2^,^3^,^4^. However, current characterization techniques lack the capability to fully dissect the complex dynamics of the pulse amplitudes and phases (or phase correlations, i.e. coherence) in real-time, continuously. On the fundamental physics front, such ability is invaluable to unravel the underlying mechanisms resulting in stochastic behaviors of the ultrafast pulse generation, which are closely linked to the intricate interactions between noise and nonlinear dynamical processes^5^,^6^. On the practical usage front, real-time monitoring and analysis of the shot-to-shot (at a rate of MHz or even GHz) amplitude/phase fluctuation of the pulses are critical for identifying the determining parameters/conditions that lead to stable and coherent pulsed lasers, which are stringently required by ultrafast applications^7^,^8^,^9^,^10^,^11^,^12^,^13^,^14^,^15^,^16^.

Primarily limited by the acquisition speed of the spectrometers, classical spectral characterization techniques can only carry out ensemble averaged measurements, which mask the subtle properties of the high-speed amplitude and phase dynamics. Meanwhile, nonlinear optical gating, and spectral shearing interferometry can be employed to retrieve the amplitude and phase of single pulses^17^,^18^,^19^,^20^,^21^,^22^. Although good sensitivity can be offered by these techniques with a trade-off of relatively long detection time, they have only been applicable to repetitive events and lack the ability to perform measurement in real-time. Toward resolving such predicaments, a new class of spectral characterization techniques, dubbed optical time-stretch, which enables shot-to-shot broadband pulse measurements has recently caught considerable attention. The core concept is to map the instantaneous spectral amplitude (or intensity) of the pulses to the time domain through group-velocity dispersion (GVD)^23^,^24^. With a sufficient amount of GVD, each single time-stretched pulse is essentially the replica of the frequency spectrum in time domain. Real-time single-shot spectral acquisition is thus simply achieved by high-speed digitizers.

While optical time stretch has opened a new avenue for spectrally-resolved single-shot characterization at an ultrahigh speed, majority of the existing effort focuses on intensity measurement and analysis^25^,^26^,^27^,^28^. Although phase retrieval from single pulses is possible with iterative computational techniques^29^, the phase accuracy is inherently limited by the time-bandwidth product, not to mention that the increased experimental complexity due to the required multiple/cascaded measurements^30^. To date, there is apparently a missing element in the optical time stretch technique that enables ultrafast broadband phase variation measurements in real-time. Tracking the dynamics of the phase or coherence is of profound interest particularly in studying the stochastic processes involved in complex ultrafast pulse generation. But evidence from direct experimental measurements still remains elusive. Especially for ultrafast pulse generation involving high nonlinearity and stochasticity, phase coherence is an important characterization metric. For instance, phase coherence of modulation instability^31^ and rogue wave formation can be dramatically influenced by the presence of deterministic seeds/triggers^10^,^32^,^33^. In other nonlinear systems that involve solitonic dynamics^34^, optical turbulence and chaotic behaviors^35^,^36^, measurements of both spectral phase coherence as well as the intensity fluctuation in real-time have also long been absent, yet are expected to provide a more complete understanding of the underlying physics.

We here report the first experimental characterization of real-time spectral coherence with a temporal resolution down to microseconds based on optical time-stretch combined Young’s type interferometry. Seemingly straightforward, our approach possesses two intriguing and under-explored features, which will be elucidated in this work. First, our approach hinges on high-speed evaluation of the measured visibility of ultrafast single-shot interferograms continuously-generated by interfering the neighboring pulses. While the evaluation of the degree of coherence is still based on ensemble of pulses, the key feature of the present technique is its capability of accessing large population of single-shot interferograms within a short time scale, thanks to the high-repetition rate operation (>10’s MHz). This is in contrast to the traditional Young’s type interferometric methods in which the spectral acquisition speed is limited by the imaging sensor employed in the classical spectrometers. Also, our technique offers a new dimension to study the two-wavelength cross-correlation map of the spectral coherence which is the cross-spectral density (CSD) function, i.e. second-order spectral coherence^37^-another feature generally absent in the current spectroscopic measurement tools. Therefore, the technique, which can simultaneously measure broadband spectral intensity in real-time, represents a powerful tool for comprehensive characterization of ultrafast pulses, in terms of tracking the intensity and coherence variation at an unprecedentedly high speed.

## Results

### Spectral coherence and CSD

We first recall the definition of spectral coherence widely adopted in the context of supercontinuum (SC) generation^6^,^38^. It is defined as the modulus of the complex degree of first-order coherence at each wavelength for a given light source


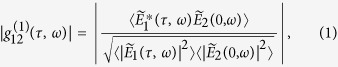


where 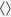 denotes the ensemble average over the independently generated optical field (single-shot pulse) pairs *E*_1_ and *E*_2_. *τ* is the time delay introduced between two fields. This definition entails the need for accessing full-field information of any arbitrary pair in a large ensemble. Such measurement is in practice still formidable with any current technologies. To date, the most practical approach to obtain the spectral coherence experimentally is Young’s type interferometry, in which single pulses interfere with the adjacent pulses. This results in the spectral interferograms which can be captured by any spectrometers. Without the ability to capture the single-shot interferograms, current spectrometers measure the large ensemble average. In this scenario of long integration time, the modulus of 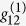 essentially corresponds to the fringe visibility observed in the interferograms. Combining optical time-stretch with Young’s interferometry, one can perform wavelength-to-time mapping of the interfering pulse-pairs via GVD, which results in single-shot interferograms captured continuously in time-domain. We can thus leverage the large ensemble of single-shot interferograms captured at a short time-scale to probe any fast dynamical variation of spectrally resolved coherence. We note that the statistical representation of any arbitrarily generated pulse-pair of optical fields is in principle not identical to that of the consecutive adjacent pulse-pairs generated by our technique. Nevertheless, such difference in pairing does not significantly influence the spectral coherence, provided that the sample size (number of pulses) is sufficiently (>200) (See Supplementary Figs S6 and S7). This can be readily achieved by our technique within microseconds, thanks to the ultrafast pulsed laser repetition rate, which is typically as high as MHz to GHz. (See the detailed validation in Supplementary Information).

More significantly, our technique allows us to experimentally estimate the CSD, which is the second-order spectral coherence, in real-time and in an ultrafast manner. Specifically, the ensemble product of the single-shot time-stretch spectral interferograms at two wavelengths closely resembles the CSD function^39^


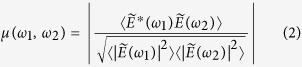



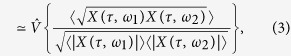


where 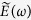 is the complex spectrum of the Fourier transform of the envelope of the slowly varying optical field, 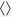 denotes the ensemble average, *X*(*τ*, *ω*) is the spectral interferogram with a temporal mismatch of *τ*, and 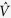 denotes as the visibility operator which computes visibility of the normalized 2-D ensemble mean of spectral interferograms. The approximation in Eq. (3) is based upon that the time delay between the neighbouring pulses is sufficiently long and the two pulses can be considered to be independent^38^,^39^. (See the detailed formulation in Method Section). We validate this connection by studying both the fs-pumped and ps-pumped SC (See Supplementary Figs S4 and S5).

### Experimental configuration

**Figure 1:**
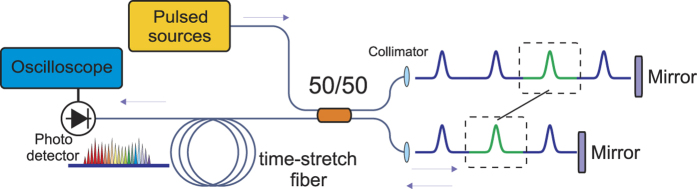
Schematic of experimental setup of the time-stretch-based Young’s delayed interferometer. The neighboring pulses are combined and interfered. Then the interfered pulses go through a 10-km long DCF after which the single-shot interferograms are mapped into the time-domain. 50/50 refers to the beam splitter with a 50:50 splitting ratio.

In contrast to the conventional configuration adopted for the static spectral coherence measurement^18^,^19^,^20^,^21^,^22^, our experimental setup consists not only a free-space Michelson interferometer with one of the arms delayed by one period of the pulse source, but also a time-stretch module after the interferometer-the enabling element for ultrafast spectral coherence measurement as shown in Fig. 1. The interfered pulses are time-stretched by a 10.7 km-long dispersion compensation fiber (DCF) with a total GVD of almost −1 ns/nm, which results in a spectral resolution of 0.1 nm (determined by the wavelength-time mapping process^23^). To exemplify the key features of the technique, we employ this setup to characterize the spectral coherence of three different pulsed laser sources with a temporal resolution down to microseconds (See Supplementary Information).

### Spectral coherence measurements of fs-pumped and ps-pumped SC generation

**Figure 2:**
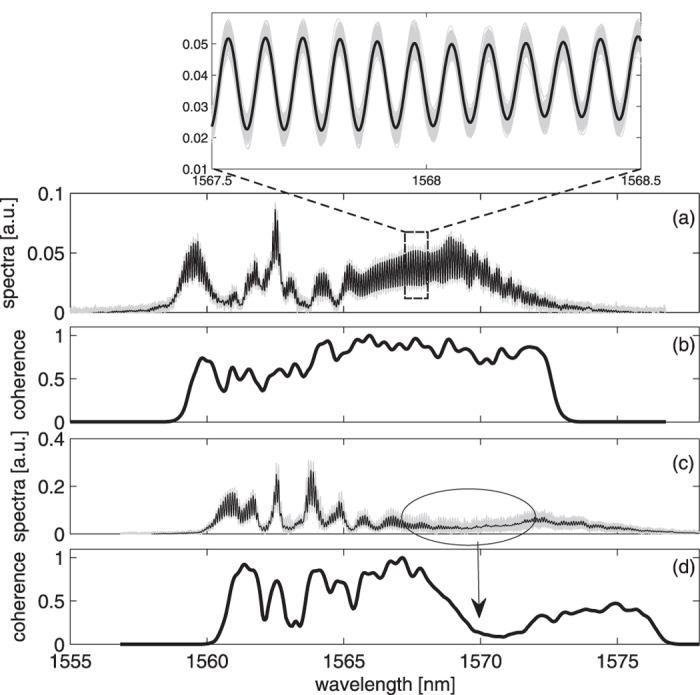
Measured spectral coherence by time-stretch interferometry. (**a**,**c**) are the experimentally observed 500 consecutive single-shot interferograms (the overlapped gray interferograms) of the SC generated by the femtosecond (780 fs) and picosecond (2.2 ps) pumps, respectively. They are captured at a rate of 46 MHz. The black interferograms are the averaged interferograms. (**b**,**d**) are the corresponding spectral coherence.

We first demonstrate the capability of our technique to observe the conventional static SC spectral coherence. Thanks to the time-stretch technique, we capture 500 single-shot interferograms for both pumping scenarios at an ultrafast rate of 46 MHz (see Fig. 2(a,c)). Note that each spectral interferogram is intrinsically generated by the interferometer, and is transformed into a time series of interferogram by optical time stretch. Again, we here focus on the degree of spectral coherence influenced by the amplitude and phase stability which can be evaluated by the fringe visibility of the interferograms. Based on the numerical study presented in Fig. S7 (See Supplementary), the ensemble size of *n* = 500 is sufficiently large to quantify the spectral coherence resembling 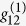 which is evaluated for a large ensemble size.

As expected, in the case of fs-pumping regime, in which the spectral broadening is dominated by self-phase modulation (SPM) and subsequent soliton fission. High spectral coherence can be clearly observed over the entire spectrum (Fig. 2(b)). On the contrary, the SC generated by ps-pumping (Fig. 2(d)) is generally less coherent in the long wavelength side, especially from 1568 nm to 1572 nm (indicated by the circle in Fig. 2(c), with the corresponding spectral coherence pointed by the arrow in Fig. 2(d)). We attribute this low coherence region to the process of the soliton formation which onsets from the noise-driven modulation instability effects when pumping a ps-pump^6^,^37^. Note that as the entire ensemble of interferograms is captured well within millisecond in the experiments reported in this work. Any bulk optomechanical vibration and drifting of the system is expected to be in the low frequency range (≪kHz), which is thus negligible in our measurements. For long-term continuous measurements, feedback stabilization of the mechanical drift can be implemented to ensure long-term mechanical stability of the interferometer.

Apart from evaluating the static coherence, we also show that time-stretch-based Young’s interferometry uniquely enables a more comprehensive, yet underexplored approach to experimentally study the CSD function, i.e. second order coherence. Again, we leverage the ensemble of the single-shot interferograms *X*(*τ*, *λ*) to compute the estimated CSD function 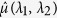. Note that *λ* = 2*πc*/*ω*, is the wavelength and *c* is the speed of light. (See Section II in Supplementary Information for details of reconstruction of CSD using the ensemble of spectral interferograms).

**Figure 3:**
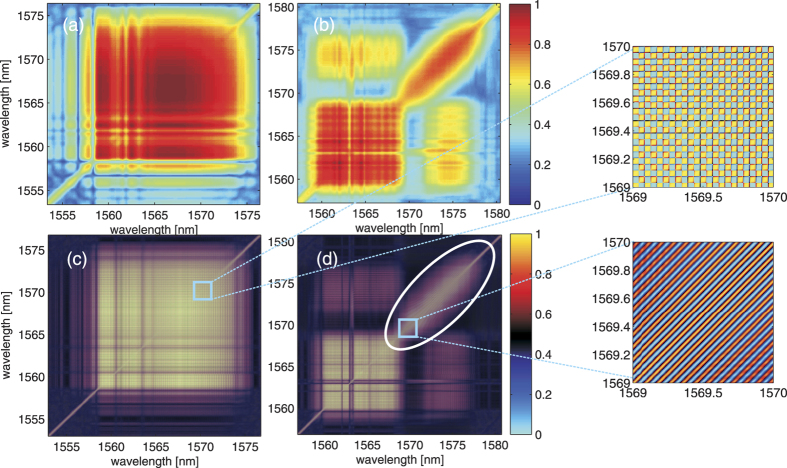
Estimated cross-spectral density function (CSD) derived from time-stretch interferometry. (**a**,**b**) are the estimated CSD 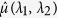 functions of SC sources in the femtosecond and picosecond pumping regimes, respectively. (**c**,**d**) are the ensemble averages of 2-D spectral interferograms 〈*X*(*λ*_1_)*X*(*λ*_2_)〉 corresponding to the subplots (**a**,**b**), respectively. Subplots on the right hand side are the zoom-in views of the highlight regions of the 2-D spectral interferograms. 500 single-shot interferograms are taken for the calculation.

Figure 3(a,b) show the estimated CSD function 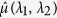 of for both ps and fs-pumping cases. In the case of fs-pumped SC, it displays a high degree of coherence correlation across the entire spectrum, manifested as an almost uniform square with the value close to unity in the map (Fig. 3(a)). It means that the spectral components are highly correlated in this ensemble, and this is consistent to the fact that the fs-pumped SC generation is primarily dominated by the deterministic spectral broadening processes. In contrast, the CSD function of the ps-pumped SC only has the high coherence correlation mainly along the diagonal line (corresponding to the self-correlation of each wavelength component), leaving a relatively low coherence along the two sides of the diagonal line of the map. This means a general incoherence and low spectral correlation across the long wavelength of SC, which is attributed to the noise-driven MI and the subsequent spectral broadening in the ps-pumped SC. Note that the map can reveal a finite spectral correlation between the pump and the soliton regions (see the top left (or bottom right) region in the map (Fig. 3(b)). There appears some line structures with slightly lower coherence along the estimate CSD function, and they can be attributed to the troughs of ripples of the spectral coherence that can be observed in Fig. 2(b,d).

More interestingly, when we study the normalized 2-D spectral interferograms 〈*X*(*τ*, *λ*_1_)*X*(*τ*, *λ*_2_)〉 instead of 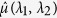, we observe that the fs-pumped SC exhibits high visibility with a sharp square-grid pattern in the long wavelength side (close to the diagonal line) whereas the ps-pumped SC shows the sheared grids or even diagonal strips (Fig. 3(c,d)). The square grids are a result of the well-defined change in visibility across the interferograms (i.e. the magnitudes of maxima and minima of the interferograms vary collectively). On the contrary, the stripe lines are the results of the self-correlation of the low-coherence long wavelength components (indicated by the white circle) in which the spectral fringes of self-correlation envelope line up with respect to the diagonal line of the CSD. Shearing of the grid represents not only the change in visibility, but also the spectral phase jitter across the spectrum. This follows the similar jittering argument used in intensity cross-correlation analysis^26^,^40^. The difference is that we focus on the phase jitter as we consider the spectral interferograms instead of the spectral amplitudes. Therefore, sheared grids in Fig. 3(d) can be interpreted as the spectral phase jitter of the new frequency components generated by the Raman induced self-frequency shift. This spectral phase jitter effectively leads to the low spectral coherence in the ensemble, as indicated by the square in Fig. 3(d).

### Simultaneous measurements of spectrally resolved intensity and coherence of mode-locked laser

**Figure 4:**
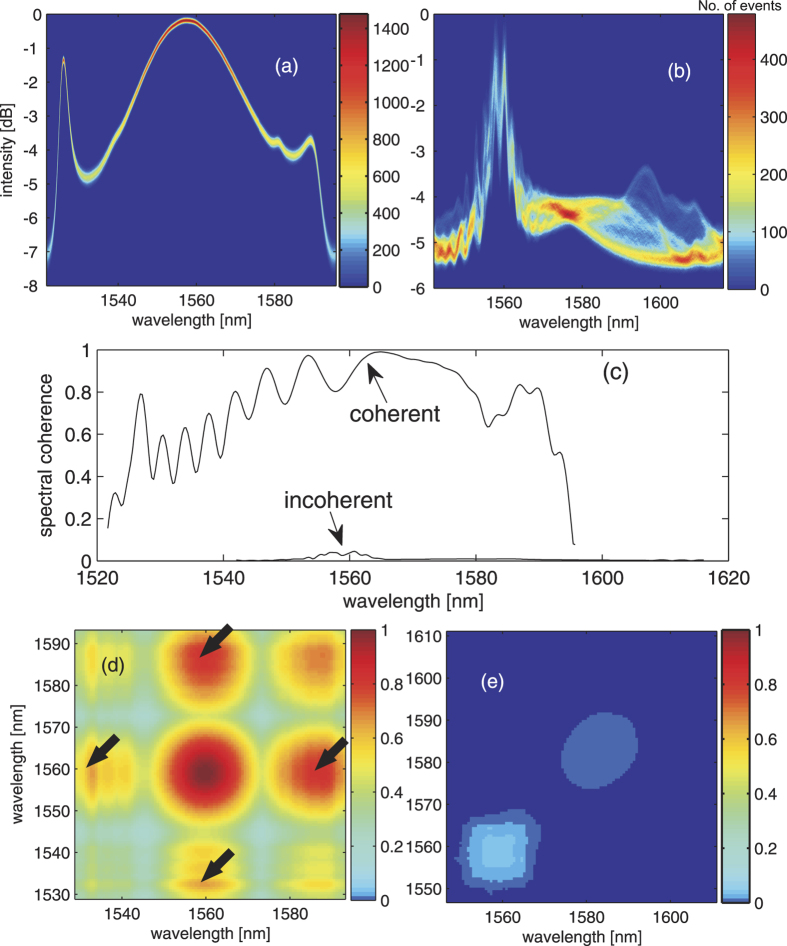
Experimentally measured CSD functions of a mode-locked laser. (**a**,**b**) are spectrally-resolved power histograms under coherent and incoherent conditions, respectively. (**c**) is the ensemble averaged spectral coherence under coherent and incoherent conditions (indicated by arrows). The black curves are the corresponding ensemble averaged of the spectral coherence. (**d**,**e**) are estimated the CSD functions of coherent and incoherent pulses, respectively. In the current setup, we respectively capture ~40,000 single-shots intensity spectra and interferograms within 0.5 ms.

We also perform a similar characterization on a mode-locked fiber laser, with the focus on demonstrating the simultaneous real-time spectral measurements of intensity as well as coherence at a fast rate of tens of MHz(See Supplementary Information for experimental setup). Simultaneous broadband spectral intensity measurement can simply be done by introducing additional detection channel which directly measures the time-stretched intensity spectra without going through the interferometer. This has minimal modification of the optical setup and is thus a handy method to measure both the dynamical variation of coherence and amplitude in real-time and continuously, with a temporal resolution down to sub-microsecond. In the current setup, we respectively capture ~40,000 single-shots intensity spectra and interferograms within 0.5 ms. The laser can be operated in either a coherent (mode-locked) or incoherent (partially mode-locked) mode by adjusting the state of polarization of the optical pulse inside the fiber cavity. In the mode-locked case, the single-shot intensity spectra, including the characteristic Kelly sidebands, are highly stable (Fig. 4(a)). It also shows a high degree of coherence across the entire lasing spectrum (evaluated based on the single-shot interferograms)-clearly validating the classical mode-locked operation (Fig. 4(c)). Moreover, we observe the reconstructed CSD of the coherent mode-locked pulses has two distinct characteristic regions showing high coherence (see the arrows highlighted in Fig. 4(d)): the stable cavity soliton (central part of the CSD), and the Kelly sidebands which are generated through the quasi-phase matched parametric process with the cavity soliton. In contrast, the partially mode-locked laser is essentially incoherent (Fig. 4(c)) even the single-shot intensity spectra are not completely stochastic (note that the main spectral shape of the lasing spectrum around 1560 nm is still discernible in Fig. 4(b)), albeit not as stable as the mode-locked case). Also, the CSD in this case is virtually zero, except showing very low value (~0.06) in the spectral region near 1560 nm, indicating that there is no obvious phase correlation between consecutive pulses. We note that such a comprehensive ultrafast pulse characterization is not only limited to the laser configuration described here. It would also be useful for investigating any laser systems in real-time that do not operate in the typical coherent mode-locked regime, but in a stochastic and partially coherent regime, e.g. the periodic or localized spatio-temporal structures observed in the long-cavity laser systems^36^. Traditionally, the behavior of such laser can only be studied by means of numerical simulation because of the lack of real-time experimental measurement techniques. Time-stretch interferometry reveals single-shot intensity spectra as well as the coherence spectra in real-time and at high temporal resolution. It is thus a unique, practical tool for in-depth experimental study of nonlinear physics in many ultrafast laser systems. Indeed, as discussed in the next section, we also demonstrate the dynamical spectral coherence monitoring of a buffered cavity laser by using time-stretch interferometry.

### Spectral coherence dynamics measurement

To further demonstrate the capability of high-speed spectral coherence variation measurement enabled by our technique, we study another type of pulsed laser source-generating optical buffered pulses within an all-fiber ring cavity, which is modulated (“addressed”) with an external mode-locked laser^41^. It is proven to be an effective all-optical approach to actively manipulate the intra-cavity multipulses in mode-locked lasers, which commonly emerge from the combination of nonlinear mechanisms in the presence of noise, e.g. spectral filtering effect, wave breaking effect and soliton peak clamping effect. As far as stable mode-locked laser development, buffered light source is of great value to control pulsing in the laser cavity. Detailed configurations can be referred to the Supplementary Information. In brief, the cavity originally emits continuous wave at 1558 nm in the absence of the addressing pulses. It in turn sustains buffered pulse oscillation when modulated, via cross-phase modulation, by an external addressing pulsed laser with a large wavelength-detune (at 1060 nm in our case)-resembling as a saturable absorber effect^42^,^43^. As the resultant modulated pattern is replicated from the addressing laser^44^,^45^ with an ultra-wideband wavelength conversion of 500 nm, this scheme is particularly useful for all-optical delay lines^46^.

**Figure 5:**
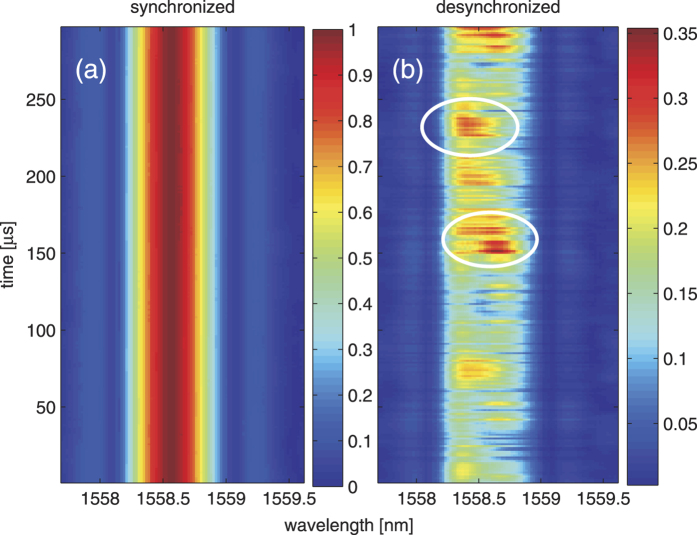
Time evolution of spectral coherence of buffered cavity. (**a**) Perfectly synchronized injection and (**b**) partially desynchronized injection. Highlighted circles show the “slow” localized structures embedded in the stochastic variation of spectral coherence.

It is expected that the coherence of the buffered pulses is strongly influenced the level of synchronization between the buffered cavity and the external addressing pulsed laser cavity, which can be manipulated by the cavity lengths. We thus measure in real-time the spectral coherence evolution of the buffered pulses in two scenarios in which (i) the cavities are perfectly synchronized; (ii) the cavities are partially desynchronized (Fig. 5(a,b)). Each measurement consists of 8192 single-shot interferograms over a time windows of 300 *μ*s. We compute spectral coherence evolution based on a moving ensemble with 256 interferograms. With an overlapped size between the neighboring ensembles of 32, the equivalent temporal resolution of the real-time spectral coherence measurement is as short as 1.2 *μ*s. This implies the system is able to probe fast spectral coherence variation close to the range of MHz.

Clearly, the perfectly synchronized buffered pulses possess a higher and more stable spectral coherence than the partially desynchronized pulses. The low spectral coherence of the desynchronized pulses is mainly attributed to “modulation” timing jitter between addressing pulses and the buffered intracavity pulses. The ultrashort time jittering manifests itself as the spectral phase shifts and consequently low spectral fringes contrast of an ensemble. While the spectral coherence of the desynchronized pulses exhibits a large amount of fluctuations along the evolving time, our technique enables us to identify an interesting feature that moderate coherence (~0.35) is localized over both spectral domain (within 0.5 nm) as well as and “slow” temporal evolution domain (over 20 *μ*s). (see the highlighted circles of Fig. 5(b)). These local structures appear in a rather stochastic manner, yet tend to persist for hundreds of cavity round-trips. To the best of our knowledge, such real-time, fast, and non-repetitive coherence variations have not been experimentally revealed by any existing (slow) spectral measurement techniques. We note that similar stochastic, localized radiations have also been observed in the partially mode-locking scenarios^36^. While it is yet to be fully understood, the availability of both spectral coherence and intensity information in real-time could bring new insights as well as experimental methods in understanding the complex (and high-speed) nonlinear processes. Notably, one could leverage on the arbitrary addressing patterns for flexible and high-precision synchronized coherence measurements, such as pump-probe studies, seeding analysis of stochastic processes in highly nonlinear systems (e.g. SC generation, partially mode-locking, and chaotic lasers).

## Discussion

Taking the advantage of its capability to quantify the high-speed broadband spectral coherence in real-time, single-shot time-stretch interferometry is particularly useful for probing the stochastic or noise-sensitive systems in which the amplitude and phase fluctuation dynamics are in short-time scale. These systems have been attracting considerable interest from the fundamental physics point of view. Notable examples include complex nonlinear intracavity dynamics in mode-locked laser, that is recently known to lead to chaotic or noise-like radiation, e.g. optical turbulence^47^,^48^,^49^; stochastic behaviors in SC generation, e.g. modulation instability, the subsequent soliton fission and rogue wave formations^50^. Indeed, our ability to experimentally probe these dynamics has been limited to the spectral intensity domain, which is not exhaustive. The technique demonstrated here enables comprehensive ultrafast pulse characterization, including not only single-shot intensity spectra, but also the coherence spectra at a sub-microsecond time scale in real-time. It should be reminded that the present technique follows the traditional concept of evaluating the degree of coherence, i.e. based on an ensemble average of the interferograms. The key feature here is the availability of the large population of interferograms (by interfering neighboring single-shot pulses) within a short time scale not achievable with any existing techniques - thanks to the ultrafast operation enabled by optical time stretch. More importantly, this attribute also allows us to experimentally evaluate the second-order spectral coherence, i.e. CSD-another feature generally missing in the current techniques. Comparing with the existing CSD measurement techniques, we highlight that our technique is able to recover the CSD without separating the measurements of the coherent and quasi-stationary parts^39^,^51^.

Given these capabilities, the technique could bring new perspectives in experimental investigations of those intricate nonlinear dynamics which have only been possible with numerical simulations. For instance, many of these effects are sensitive to input noise or other external perturbation. Hence, one can implement synchronized pump-probe, feedback, seeding/triggering measurements in which the seed/trigger can be arbitrarily engineered (in terms of temporal/spectral width, power, and spatio-temporal patterns) to probe and even manipulate those phenomena. Using time-stretch interferometry can readily reveal the corresponding real-time rapid responses in both spectral coherence as well as intensity variations. More practically, deeper understanding of these underlying processes could provide insight into how to manipulate them and thus pave the way for developing highly stable (shot-to-shot), low-noise pulsed lasers, especially valuable for applications requiring high temporal stability, such as ultrafast imaging and spectroscopy^2^. Note that seeded/triggered SC has already been studied with time-stretch spectral intensity measurements, especially in the context of “stimulating” or “inhibiting” rogue wave formation^10^,^52^, which give clues for SC enhancement or suppression^28^, e.g. the transient of the seeding process, or the influence by any temporal modulation of the seed. Again, the dynamics of spectral coherence have not been fully characterized in many of these highly nonlinear SC generation processes.

We thus here mainly demonstrate the spectral coherence measurements of ultrafast laser pulses generated through the nonlinear processes. A well-known example, as demonstrated here, is the ps-pumped SC measurement in which the spectral phase after the nonlinear broadening is undeterministic, and the generation of new frequency components is dominated by the background noise. In principle, this technique can also be extended to other pulsed laser systems. Again, it should be noted that the present technique is not meant to measure the single-shot intra-pulse spectral phase profile, which can be captured by the techniques based on nonlinear optical gating, spectral shearing interferometry or other pump-probe techniques. Nevertheless, our technique is particularly advantageous for applications that require monitoring of non-repetitive high-speed dynamics of spectral phase coherence as well as spectral intensity continuously in real-time.

In summary, we have presented a technique, based on time-stretch Young’s interferometry, which uniquely provides access to single-shot interferograms from which real-time spectral coherence dynamics of ultrafast laser sources can be evaluated experimentally with a temporal resolution down to microsecond scale. Having validated the measured spectral coherence by comparing it with the common 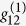 definition, we have demonstrated that the technique not only can quantify the static coherence (as demonstrated with the SC generation), but also unveil the high-speed spectral coherence fluctuation (as exemplified by a buffered cavity pulsed source) on the order of MHz. Furthermore, the large ensemble acquired by the single-shot measurement provides us sufficient information to further reconstruct the CSD function that cannot be achieved by the traditional coherence measurement techniques. The fact that the technique can also measure broadband spectral intensity in real-time makes it a handful and comprehensive toolset to gain insight into the spectral coherence dynamics of a plethora of optical ultrafast lasers as well as nonlinear optical effects, such as rogue wave formation^5^, modulation instability^31^, solitonic dynamics^34^, as well as optical turbulence and chaotic behaviors in pulse laser^35^,^36^.

## Methods

We here establish a two-frequency correlation function of the optical fields based on the ensemble of the spectral interferograms, each of which is measured by our technique in real-time. And this function can be adopted to estimate the cross-spectral density (CSD) function. Since it is technically difficult to obtain the full information of the individual optical field, our estimate of CSD relies on the normalized ensemble mean of the cross product of the spectral interferogram (i.e. 2-D spectral interferogram) as


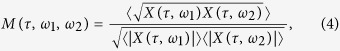


where 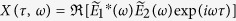 is the spectral interferogram by interfering two independent optical pulses with their corresponding spectra 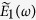 and 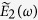, and exp(*iωτ*) is the phase term of the temporal mismatch between two pulses which also determines the oscillation frequency of the spectral interferogram. In this study, we can treat the interfered two pulses as a single field whose amplitude and the phase can be manifested by the cross term *X*(*τ*, *ω*). Thus, any shot-to-shot phase variation of a frequency component links directly to the change of fringe pattern of the spectral interferogram. We further take the square root of the *X*(*τ*, *ω*) to account for the correlation of the field rather than the intensity. Effectively, for a highly coherent broadband source, small amplitude and phase fluctuations can result a uniform chessboard pattern (i.e. high visibility), and it implies that the field at any two frequency are highly correlated (either positive or negative); while large amplitude and phase fluctuation can result a smoother chessboard pattern and implies low correlation between two fields. Finally, we evaluate our estimate of CSD 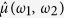 through direct calculate the visibility of the normalized 2-D spectral interferogram as


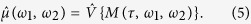


where 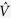 is the visibility operator. Here we stress that the estimated CSD should be distinguished from the coherent part 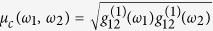 of the CSD defined in ref. 39. In this reference, *μ*_*c*_(*ω*_1_, *ω*_2_) is based on the product of the first-order coherence function, whereas our estimated CSD 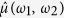 is based on the ensemble average of the product of the spectral interferograms and it can directly recover the second-order spectral coherence without separating the measurements for retrieving the coherent and quasi-stationary parts, as proposed in ref. 39.

To further verify our estimate of the CSD, we perform numerical simulations and compare the CSD *μ*(*ω*_1_, *ω*_2_) and the estimate of CSD 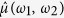 using fs-pumping and ps-pumping SC. See Supplementary Information.

## Additional Information

**How to cite this article**: Xu, Y. *et al.* Ultrafast measurements of optical spectral coherence by single-shot time-stretch interferometry. *Sci. Rep.* **6**, 27937; doi: 10.1038/srep27937 (2016).

## Supplementary Material

Supplementary Information
