# Supplementary material for: Ultrafast measurements of optical spectral coherence by single-shot time-stretch interferometry
Source: Sci Rep. 2016 Jun 13;6:27937. doi: 10.1038/srep27937 (PMC4904794; doi:10.1038/srep27937)
Supplement: Supplementary Information [file srep27937-s1.pdf]

SUPPLEMENTARY INFORMATION

Ultrafast measurements of optical spectral coherence by single-shot time-stretch interferometry

Yiqing Xu, Xiaoming Wei, Zhibo Ren, Kenneth K. Y. Wong, and Kevin Tsia

I. Experimental Setup

The interfered pulses are time stretched by a 10.7 km-long dispersion compensation fiber (DCF) with a total GVD of -932 ps/nm at 1563.8 nm. The stretched temporal fringes, i.e. the single-shot interferograms, are then detected by a 10 GHz photodiode and a real-time oscilloscope (80 GSa/sec). The fringe period of the interferogram is adjusted such that the system has enough temporal resolution to resolve the fringes and at the same time, the fringe density over the spectral envelope is sufficiently high to retain the overall spectral feature. The SC is generated by launching a fiber mode-locked laser pulsed pump (centered at 1563.8 nm with a repetition rate of 46 MHz) into a 2-km long dispersion shifted fiber (DSF) with the zero dispersion wavelength around 1550 nm. The linewidth of the pump pulse is controlled by a linewidth tunable filter. The filtered pump pulse is pre-amplified by an erbium doped fiber amplifier (EDFA) before the DSF. Using the tunable filter, we are able to manipulate SC in two different pumping regimes (as shown in Figs. 2 and 3 of the main text): (i) picosecond (2.2 ps) and femtosecond (780 fs) pulsed pump.

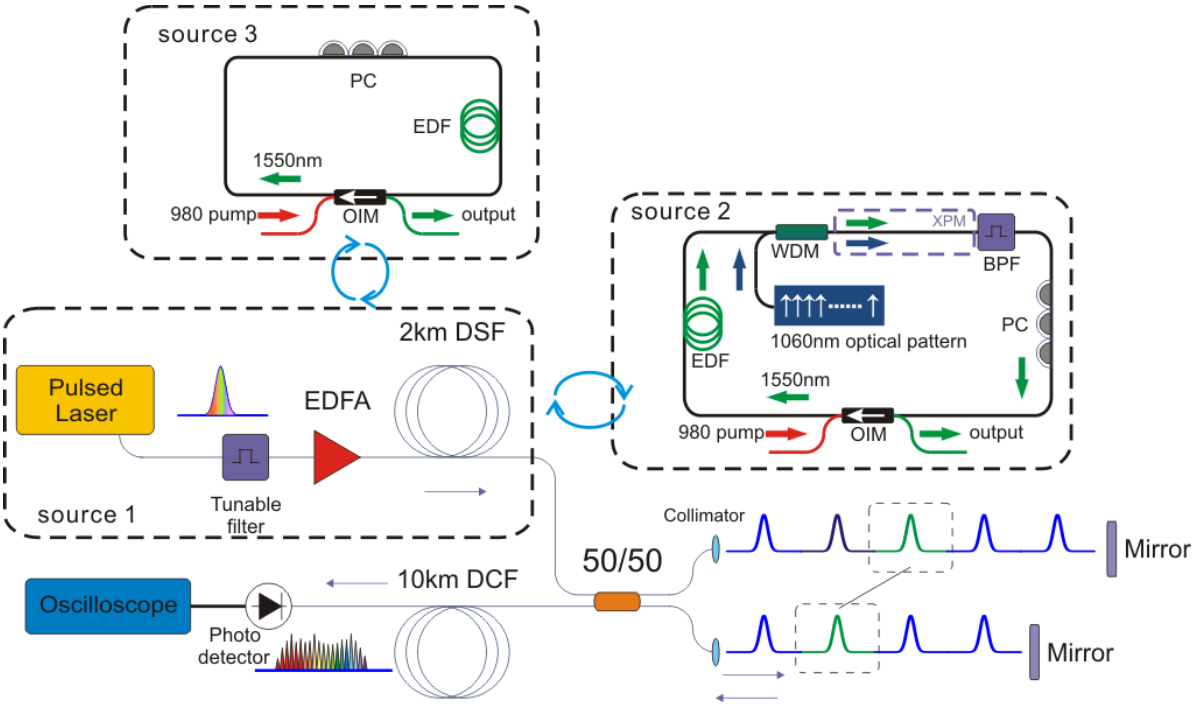

Fig. S1. Schematic of experimental setup of the time-stretch-based Young's delayed interferometer. Three different pulsed sources investigated in this work are shown here. Detailed schematic diagram of each source is shown in the respective dashed box. The neighboring pulses are combined and interfered. Then the interfered pulses go through a 10-km long DCF after which the single-shot interferograms are mapped into the time-domain. 50/50 refers to the beam splitter with 50:50 splitting ratio. EDFA: Erbium-doped fibre amplifier, DSF: dispersion shifted fiber, DCF: dispersion compensation fiber, OIM: optical integrated module, PC: polarization controller, BPF: bandpass filter, EDF: Erbium-doped fibre,WDM: wavelength division multiplexer.

Buffered cavity laser. This light source, used for the coherence dynamics measurement (Fig. 5 in the main text), is optical buffered pulses which are generated by an all-fiber ring cavity buffer with an external addressing mode-locked laser, as shown in Fig. S1 (Source 2). For more details about this cavity buffer and addressing laser, please refer to Ref.[1, 2]. The inline polarization controller (PC) before optical integrated module (OIM) is employed to adjust the state of polarization of the optical field inside the all-fiber ring cavity, which together with the polarization-sensitive OIM sustains the buffered pulse oscillation after excited by an external pulse laser with a wavelength far beyond the lasing wavelength of the fiber cavity, i.e. promotes the artificially saturable absorber effect[3, 4]. The buffer cavity is adjusted to emit continuous-wave (CW) around 1558 nm without the addressing pulses. The CW output from the EDF is combined with the addressing signal pulse from an external pulse laser at 1060 nm for the excitation through a fiber-based WDM. The 1558 nm CW wave is "modulated", via cross-phase modulation (XPM), to be the pulse pattern the same as that of the addressing beam[5, 6]. For the purpose of testing the coherence of the buffered pulses, we control the coherence of the buffered pulse by adjusting the synchronization between the length of the buffered cavity and addressing pulsed laser cavity.

Fiber mode-locked laser. This is used for demonstration shown in Fig. 4 of the main text. The detailed setup of the partially mode-locked laser can be found in Ref.[7] (see Source 3 in Fig. S1). Similar to the buffered cavity laser source, the broadband pulses are generated by the additive pulse mode-locking technique associated with a fiber-based OIM component mentioned above. The polarization controller is a key element to set the operational condition to either stable or unstable mode. The mode-locked pulses were extracted out from the ring cavity via the tap port of the OIM. The total cavity length is ~2.6 m with a repetition rate of 80 MHz. The total fiber dispersion is now reduced to -0.166 ns/nm to avoid the overlapping of the time-stretched pulses. We plot in Fig. S2 (a) and (b) the output spectra of this source (measured by the conventional optical spectrum analyzer (OSA)) under the stable (coherent) and unstable (incoherent) mode-locking conditions.

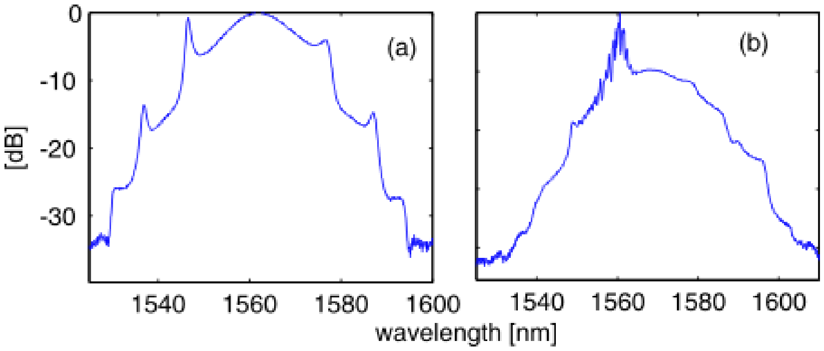

Fig. S2 Output spectra (measured by the conventional OSA) under (a) stable and (b) unstable conditions of the mode-locked laser.

II. Formulation of cross spectral density function of supercontinuum generation

In this section, we outline the procedure the two-frequency cross spectral density function (CSD) of the supercontinuum generation (SC) retrieved from the intensity of the ensemble interferograms. We firstly numerically simulate 1000 runs of optical pulse (Gaussian pulse profile) propagated along a 20 m photonic crystal fiber (PCF) with the nonlinear Schrödinger equation (NLSE):

$$\frac{\partial A}{\partial z} = \sum_{k\geq 2} \frac{i^k}{k!}\beta_k \frac{\partial^k A}{\partial t^k} + i\gamma A(z,t) \int_{-\infty}^{+\infty} R(t')|A(z,t-t')|^2 dt', \quad (1)$$

where $A(z,t)$ is the envelope of the slowly varying optical field, β_k are the dispersion coefficient, γ is the nonlinear coefficient of the PCF, and $R(t)$ is the Raman response function contained the instantaneous and Raman delay attributed to the nonlinearity. Please refer to Ref.[8] for the details of the simulation parameters. SC generated in two different pumping regimes-ps pumping (1.2 ps) and fs pumping (400 fs) are investigated. The simulated output spectra at the end of the PCF for these two scenarios are shown in Fig. S3.

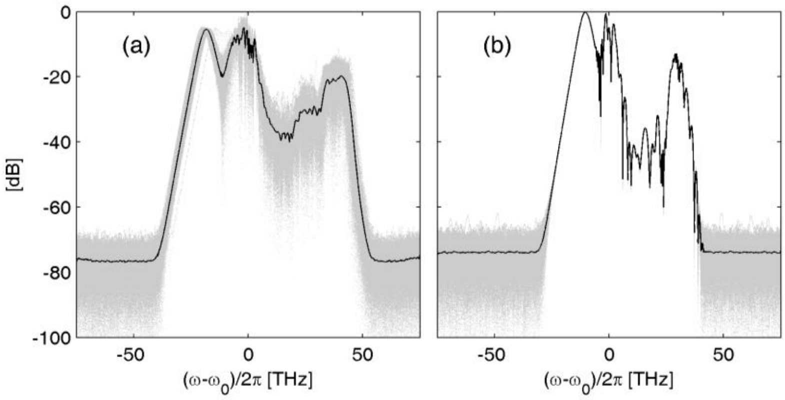

Fig. S3 The ensemble of simulated SC of 1000 runs. (a) and (b) are the SC generated by ps-pumping and fs-pumping, respectively. The gray dots are the ensemble of the spectra, and the black curves are the spectra of the ensemble average.

To demonstrate how to recover the CSD function with the ensemble of spectral interferograms, we compute the estimated CSD $\hat{\mu}(\omega_1, \omega_2)$ using the ensemble of the intensity profiles of the spectral fringes $I_n(\omega, \tau) = |\mathcal{F}[A_1(t) + A_2(t + \tau)]|^2$ obtained from the 1000 numerical simulation runs of SC, and compare the directly calculated CSD function $\mu(\omega_1, \omega_2)$ with the full field spectra $\tilde{E}_n(\omega)$ using Eq. (2) in the main manuscript. Following the steps indicated in Fig. S4, we plot in Fig. S4 (a) and (b), the ensemble of the spectral interferograms of SC generated by a ps-pump, and the cross-correlated interferogram $X(\tau, \omega_1)X(\tau, \omega_2)$, respectively. Finally, we calculate the recovered CSD function $\hat{\mu}(\omega_1, \omega_2)$ based on the visibility of the cross-correlation map of 2-D spectral interferograms using Eq. (3), and plot in Fig. S4 (d). By defining a proper moving window over the map, the visibility of 2-D spectral interferogram can be simply extracted from the amplitude of the frequency component of the 2-D FFT that corresponds to frequency of the spectral fringes. Compared with the original CSD function $\mu(\omega_1, \omega_2)$ that shown in Fig. S4 (c), it can be clearly seen that the recovered CSD $\hat{\mu}(\omega_1, \omega_2)$ resembles the key features of the original CSD function $\mu(\omega_1, \omega_2)$.

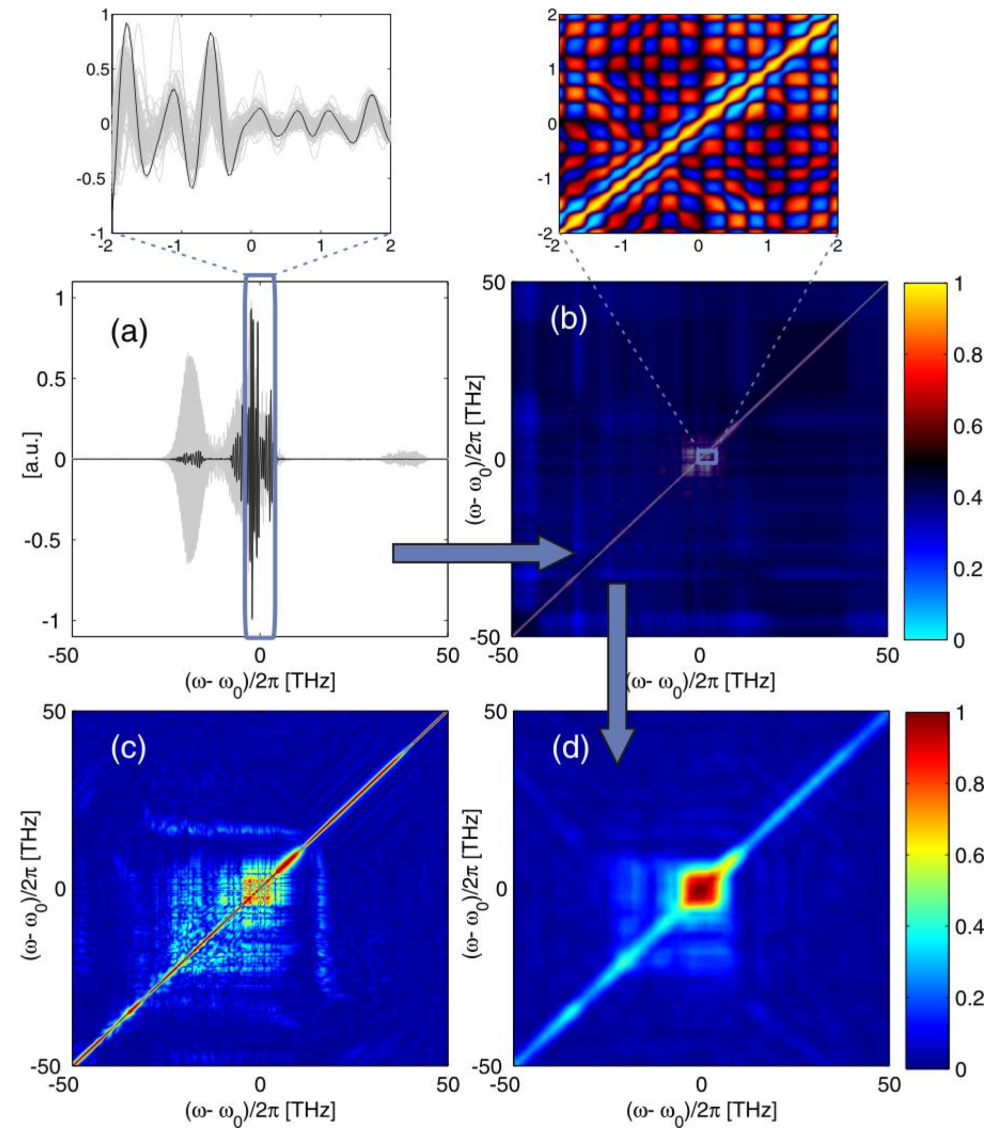

Fig. S4 (a) The ensemble of the spectral interferograms $X(\tau,\omega)$ (grey curves) and the ensemble average of the $\langle X(\tau,\omega)\rangle$ (black curve). (b) The normalized 2-D spectral interferogram calculated based on Eq. (6). (c) The CSD function $\mu(\omega_1,\omega_2)$ of the ps-pumped SC calculated using Eq. (4). (d) The estimate of the CSD function $\hat{\mu}(\omega_1,\omega_2)$ based on the visibility of the ensemble average of 2-D spectral interferogram in (b).

The loss of fast variation feature in the CSD function is attributed to the overlapping between the fringe period and the fast spectral variation. Experimentally, this can be simply overcome by increasing the total dispersion of the time-stretch process and increasing the bandwidth of the detection system. To further validate the recovered CSD function, we also plot in Fig. S5 $\mu(\omega_1,\omega_2)$ and $\hat{\mu}(\omega_1,\omega_2)$ of the SC generated by fs pumping. As we expected, Fig. S5 (a) shows a series of highly consistent spectral interferograms which produces a uniform 2-D spectral interferogram. Consequently, the recovered CSD function in Fig. S5(d) can well restore the feature of the original CSD function of the fs-pumped SC in Fig. S5(c)

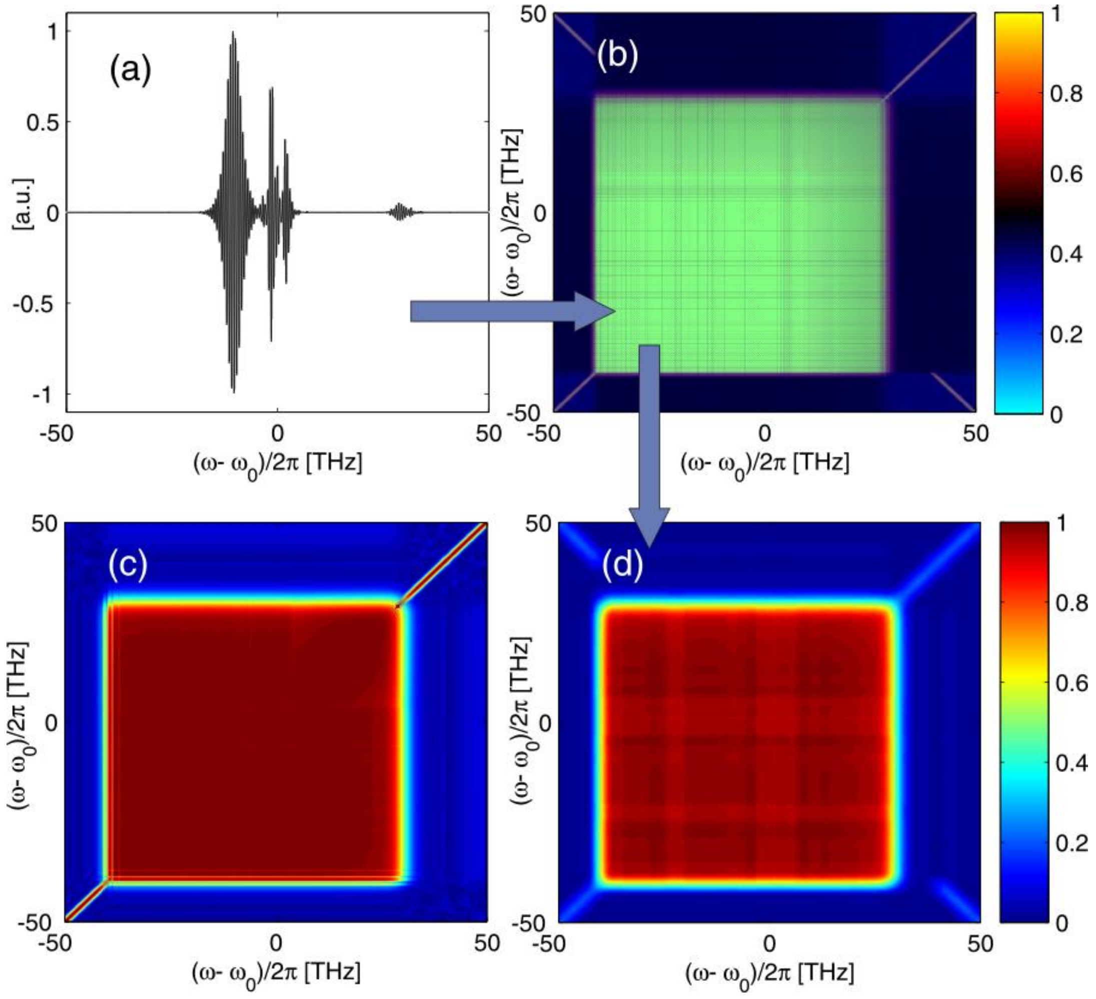

Fig. S5 fs-pumped SC (a) An ensemble of raw spectral interferograms (b) Ensemble average of the 2-D spectral interferogram (c) The CSD function $\mu(\omega_1, \omega_2)$ (d) The recovered CSD function $\hat{\mu}(\omega_1, \omega_2)$.

III. Spectral coherence measurements by OSA ensemble average

We compare the spectral coherence measured by our technique and by traditional ensemble average method with optical spectrum analyzer. We plot in Fig. S6 (c) and (d) the experimentally measured spectral coherence of the fs and ps puming SC which is originally from Fig. 3 (b) and (d) in the main text. We also measure the spectral coherence of the SC sources using an optical spectrum analyzer, and plot them in Fig. S6 (a) and (b).

As we can see, the spectral coherence measurements by OSA and time-stretch interferometry agree well with each other. We attribute the slight discrepancy between these two techniques to the long term stability of the interferometer setup, as mechanical vibration has a larger influence on the OSA measurement in which the acquisition time is in second order.

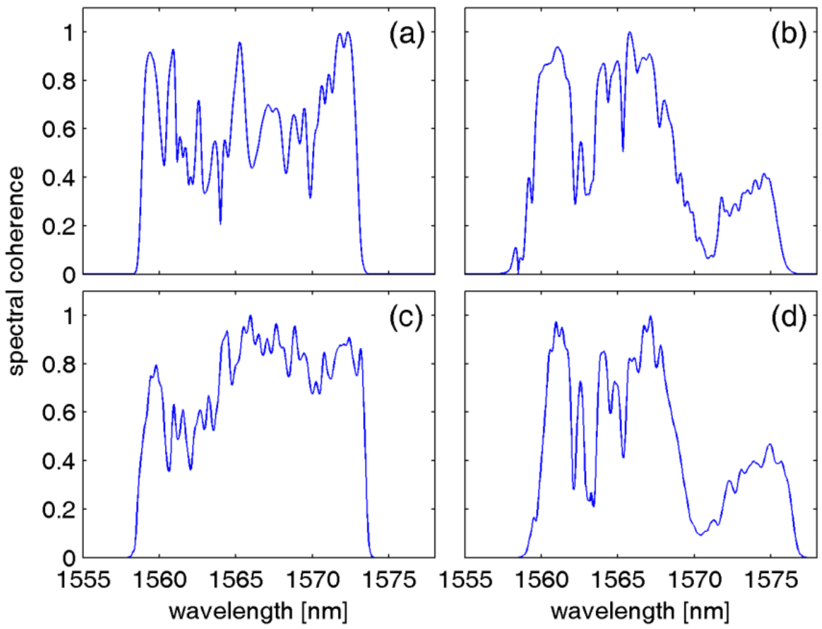

Fig. S6 (a) and (b) are, respectively, the spectral coherence of fs-pumping and ps-pumping SC generated measured by OSA. (c) and (d) are, respectively, the spectral coherence of fs-pumping and ps-pumping SC generated measured by the interferometry time-stretch setup.

IV. Statistics difference between $g_{12}^{(1)}$ and g_{Young}

The statistical representation of any arbitrarily generated pulse-pair of optical fields is in principle not identical to that of the consecutive adjacent pulse-pairs generated by the Young's interferometer. This difference stems from that the numerically simulated pulse-pairs are taken from all possible combinations of any two pulses, whereas the sequence and thus the pairing of the pulses has been determined in practical measurements, assuming each pulses are independent. Vaguely acknowledged in the literature, such difference in pairing does not significantly influence the spectral coherence. In order to validate the method of measuring spectral coherence based on time-stretch Young's delayed interferometry, we here put into context to study the spectral coherence based on Young's interferometry (denoted as g_{Young} and the original definition of $g_{12}^{(1)}$. The two definitions are differed by the pairing sequence and combination as described earlier. We investigate the difference between these two approaches using N = 1000 runs SC generation pulses at the output of the highly nonlinear fiber under the femtosecond and picosecond pulse pumping condition generated in Section II. Based on these ensembles, we compute the least square differences between the spectral coherence obtained from a finite number of ensemble (n < N) and the overall ensemble (N = 1000) for both $g_{12}^{(1)}$ and g_{Young}. $g_{12}^{(1)}$ is calculated by randomly choosing 1000 pairs of pulses from the ensemble, while g_{Young} is calculated by pairing the neighbouring

pulses from a randomly generated pulse sequence. We can observe that both cases converge in the same fashion as the ensemble size n approaches N without significant difference (Fig. S7 (a) and (b)). The calculated $g_{12}^{(1)}$ and g_{Young} are generally consistent with each other, as illustrated in Fig. S7 (c) - (h).

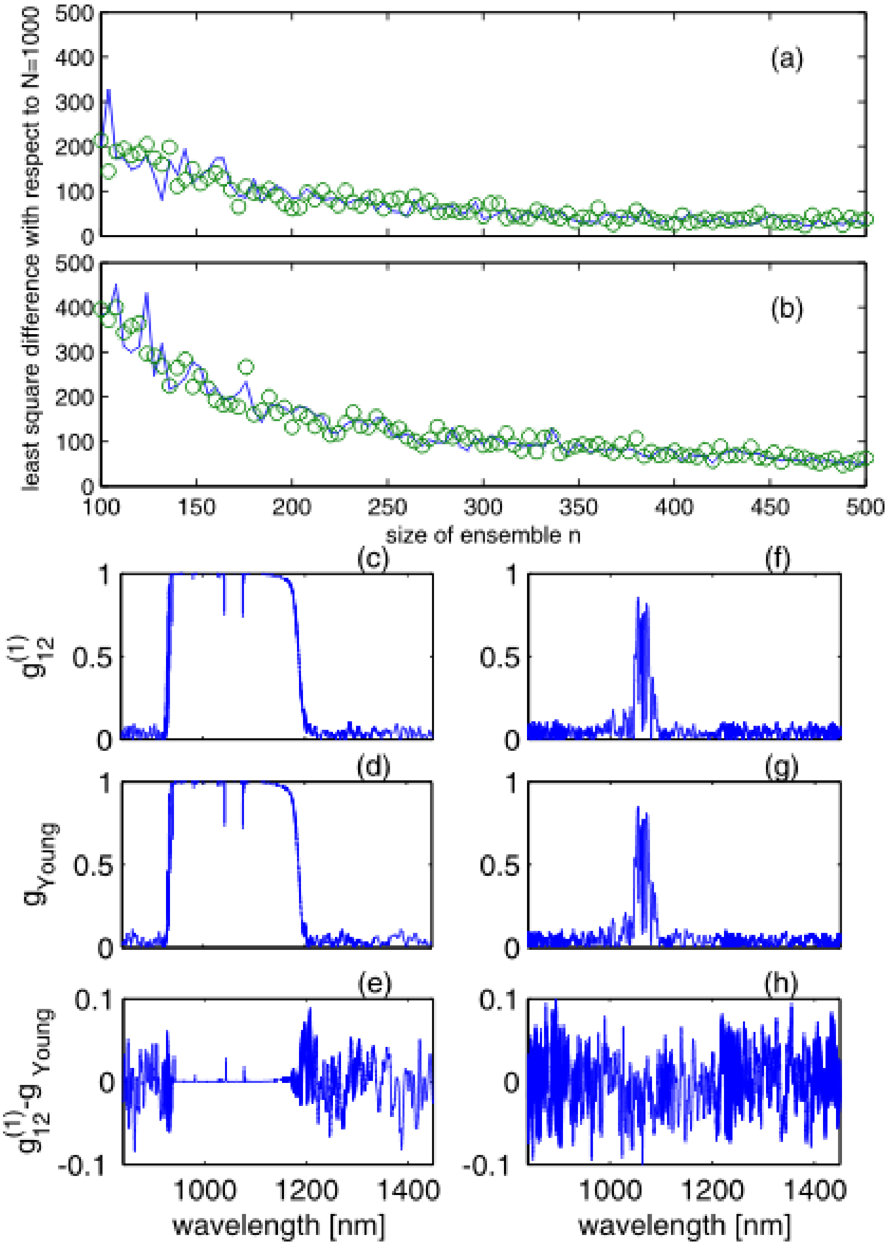

Fig. S7 (a) and (b) are, respectively, the least square difference as a function of ensemble size for fs-pumping and ps-pumping SC. Blue curves are $g_{12}^{(1)}$, and green circles are g_{Young}. (c) - (e) and (f) - (h) are $g_{12}^{(1)}$, g_{Young} and $g_{12}^{(1)} - g_{Young}$ for fs-pumping and ps-pumping SC.

V. Computation issue of spectral coherence and CSD

Time-stretch interferometry acquired the spectral data at a rate of 80 GSa/s. Taking a measurement window of 0.5 ms results in a data size of 320 Mbytes (considering the 8-bit digitizer). While the measurements are performed in real-time, the data processing (coherence, and CSD calculation) is done off-line by a personal computer. We note that "real-time" here is referred to the data acquisition that is the critical element for capturing non-repetitive information. Real-time processing is indeed possible when parallel digital signal processing based on field-programmable gate array (FPGA) and graphic processing unit (GPU) is employed for hardware acceleration. They have been widely adopted in applications where on-line processing of enormous data is critical, such as real-time ultrafast optical imaging[9].

Supplementary references

1. Wei, X. *et al.*, Coherent Laser Source for High Frame-Rate Optical Time-Stretch Microscopy at 1.0 μm. *IEEE J. Sel. Topics Quantum Electron.*, **20**, 384-389 (2014).
2. Wei, X. *et al.*, Pulsing Manipulation in a 1.55 μm Mode-Locked Fiber Laser by a 1.0 μm Optical Pattern. *IEEE Photon. Technol. Lett.* **27**, 1949-1952 (2015).
3. Agrawal, G. P., *Nonlinear Fiber Optics* (Academic Press, 2012).
4. Chong, A., Buckley, J., Renninger,W.,Wise, F., All-normaldispersion femtosecond fiber laser. *Opt Express* **14**, 10095-10100 (2006).
5. Agrawal, G. P., Modulation instability induced by cross phase modulation. *Phys. Rev. Lett.* **59**, 880-883 (1987).
6. Leo, F. *et al.*, Temporal cavity solitons in one-dimensional Kerr media as bits in an all-optical buffer. *Nat. Photonics* **4**, 471-476 (2010).
7. Wei, X. *et al*. Breathing laser as an inertia-free swept source for high-quality ultrafast optical bioimaging. *Opt. Lett.* **39**, 6593–6596 (2014).
8. Qiu, Y., Xu, Y. Q., Wong, K. K. Y., Tsia, K. K., Enhanced supercontinuum generation in the normal dispersion pumping regime by seeded dispersive wave emission and stimulated Raman scattering. *Opt. Commun.* **325**, 28-34, (2014).
9. Goda, K., Tsia, K. K., Jalali, B., Serial time-encoded amplified imaging for real-time observation of fast dynamic phenomena. *Nature* **458**, 1145-1149 (2009).
